# Acetone extracts of Berberis *vulgaris* and *Cornus mas* L. induce apoptosis in MCF-7 breast cancer cells

**DOI:** 10.55730/1300-0144.5715

**Published:** 2023-09-09

**Authors:** Burcu USLU, Mustafa YAMAN, Tuba ÖZDEMİR SANCI, Mustafa GÜNGÖRMÜŞ, Çağla Zübeyde KÖPRÜ, Fatma Esra GÜNEŞ

**Affiliations:** 1 Department of Nutrition and Dietetics, Faculty of Health Sciences, Yüksek Ihtisas University, Ankara, Turkiye; 2 Department of Nutrition and Dietetics, Faculty of Health Sciences, Istanbul Sabahattin Zaim University, İstanbul, Turkiye; 3 Department of Histology and Embryology, Faculty of Medicine, Ankara Yıldırım Beyazıt University, Ankara, Turkiye; 4 Central Research Laboratory Application and Research Center, Ankara Yıldırım Beyazıt University, Ankara, Turkiye; 5 Department of Basic Sciences, School of Dentistry, Ankara Yıldırım Beyazıt University, Ankara, Turkiye; 6 Department of Histology and Embryology, Faculty of Medicine, Yuksek Ihtisas University, Ankara, Turkiye; 7 Department of Nutrition and Dietetics, Faculty of Health Sciences, Istanbul Medeniyet University, İstanbul, Turkiye

**Keywords:** Apoptosis, *Cornus mas* L., *Berberis vulgaris*, cytotoxicity, breast cancer

## Abstract

**Background/aim:**

This study aimed to determine the proliferation and apoptotic effects of extracts from *Cornus mas* L. and *Berberis vulgaris* fruits on human breast cancer cells (MCF-7).

**Materials and methods:**

The *Cornus mas* L. and *Berberis vulgaris* fruits, which constitute the herbal material of the study, were turned into 80% acetone extract after washing. The total phenolic content in *Berberis vulgaris* fruit extracts was determined calorimetrically using Folin-Ciocalteu reagent. The spectrophotometric method was used to determine the total flavonoid amount of the extracts. In order to measure the antioxidant capacity of *Cornus mas* L. and *Berberis vulgaris* fruits and extracts, DPPH Radical Scavenging Power test and Cu (II) ion reducing antioxidant capacity method were applied. Cell viability rates were determined by the XTT method. Flow cytometric measurement was performed to examine the apoptotic role of the extracts in the cell by using the Annexin-V/7-AAD commercial kit.

**Results:**

According to the data, *Berberis vulgaris* fruit extract appeared more effective on MCF-7 breast cancer cells in both 24 and 48 hours of exposure. Analyses made to examine the phenolic component and antioxidant capacity properties of the fruits used in the study and the results we encountered when we exposed the cell were found to be compatible with each other. Annexin-V/7-AAD method showed that the apoptotic effects of the extracts in 48 hour exposures were more effective.

**Conclusion:**

It has been determined that *Cornus mas* L. and *Berberis vulgaris* fruits, which are rich in phenolic components with high flavonoid content and high antioxidant capacities, support the apoptosis of cancer cells.

## 1. Introduction

Despite the increase in conventional cancer treatment options, millions of patients suffer from cancer-related deaths every year [1]. According to the Global Cancer Observatory (GLOBOCAN) 2018 data, 18.1 million new cancer cases were seen and 9.6 million people died from cancer. According to the same data, the most common cancer types are lung (2.1 million), breast (2.09 million), large intestine (1.8 million), prostate (1.3 million) and stomach (1 million) cancers, respectively [2]. Despite advances in screening, treatment, and surveillance that have improved patient survival rates, breast cancer is still the most frequently diagnosed type of cancer and the second leading cause of cancer death among women. Breast cancer is a heterogeneous disease that is genetically based and affected by external stimuli [3]. Identification of modifiable risk factors may contribute to the development of prevention strategies aimed at reducing the incidence of breast cancer and preventing the development of more aggressive types. Breast cancer risks include environmental factors, breastfeeding, age at first live birth, parity, diet, body weight, physical activity, and alcohol consumption [4]. Diet and physical activity are two modifiable lifestyle components that can help reduce breast cancer incidence and therefore mortality. According to the World Cancer Research Fund, increasing the consumption of fruits, vegetables and whole grains, as well as limiting the consumption of red and processed meat can reduce the risk of cancer [2]. Dietary polyphenol-based therapeutic approaches are of interest as therapeutic options against chronic inflammatory diseases due to their efficacy and nontoxic nature. Recent research has aimed to discover new compounds and therapeutic approaches to reduce cancer cell survival, angiogenesis, proliferation, and metastasis. Studies have shown that the use of special phytochemical compounds and flavonoids is promising in the treatment of cancer. *Cornus mas* L. berries have a number of active ingredients, such as phenolic compounds, vitamin C, iridoids, flavonoids and anthocyanins, and an abundant source of bioactive molecules, especially polyphenols and iridoids. The biological characteristic of *Berberis vulgaris* has been attributed to the high content of isoquinoline alkaloids in the fruit and root. This herbal medicine shows different effects, including antioxidant, antiinflammatory and hypotensive activity [5]. *C. mas* and *B. vulgaris*, grown in certain regions of our country, are of particular interest with their significant amounts of phenolic compounds and vitamins. They exhibit a wide variety of biological and pharmacological properties and can be a promising resource in the prevention and treatment of breast cancer.

This study aims to increase the knowledge on the effect of extracts obtained from *C. mas* and *B. vulgaris* fruits, among cherry species rich in anthocyanins, which are found in nature. In particular, the aim is to understand how such a highly cultivated species can affect human breast cancer cells (MCF-7).

## 2. Materials and methods

### 2.1. Plant material and preparation of extracts

*B. vulgaris* and *C. mas* fruits, which were used as research material, were collected from the mountainous Pınarbaşı district of Kastamonu in August 2020, weighing 1 kg. After washing the *C. mas* and *B. vulgaris* fresh fruits, which constitute the herbal material of the study, 20 g each was weighed and 80% acetone (1:2, w/v) was added. It was then extracted for 5 min using a Waring blender. Extracted samples were filtered under vacuum through a #2 Whatman filter paper with the aid of a Buhner funnel. The rotary evaporator was kept at 45 °C until the acetone had evaporated. Then, the volume was completed up to 50 mL with water and stored at −80 °C. [6].

### 2.2. Determination of total phenolic content (TPC)

Total phenolic content in *C. mas* and *B. vulgaris* fruit extracts were determined colorimetrically using Folin-Ciocalteu reagent. First, 0.5 mL of fruit extracts were taken and then 7 mL of distilled water and 0.5 mL of Folin-Ciocalteu reagent were added to it. Afterward, it was incubated for 3 min by mixing before 2 mL of 2% Na_2_CO_3_ was added and mixed. The resulting mixture was kept in a water bath at 25 °C for 1 h. The absorbances of the samples were determined at 765 nm. Linearity ranges of total phenolic content were selected between 10 and 100 mg/L GAE using 5 calibration levels [7].

### 2.3. Determination of total flavonoid content (TFC)

The spectrophotometric method was used to determine the total flavonoid amount. To achieve this, 1-mL samples were placed in a 10-mL glass bottle, where 5 mL of distilled water and 0.3 mL of 5% NaNO_2_ were added and mixed. After 5 min, 0.6 mL of 10% AlCl_3_.6H_2_O and 2 mL of 1 mol L^−1^ NaOH were added and the total volumes were made up to 10 mL with distilled water separately. Calculations were performed by preparing a standard curve with catechin and measuring the sample homogenate at a wavelength of 510 nm using a spectrophotometer. [8]. Linearity ranges of total flavonoid content were selected between 10 and 100 mg/CE.100 mL^−1^ using 5 calibration levels.

### 2.4. Antioxidant activity determination

#### 2.4.1. Dpph

The DPPH method is based on measuring the scavenging effects of antioxidants on the DPPH radical, which is a stable free radical. It gives maximum absorption at 517 nm. By adding antioxidant to ethanol or methanolic DPPH solution, a decrease in absorbance occurs and the color of the radical changes from red to yellow with the presence of antioxidants. This method is known as an easy and valid method to evaluate the radical scavenging abilities of antioxidants. In the DPPH test, a methanolic DPPH solution was first prepared at a concentration of 0.004%. Next, separate containers were used to prepare *Cornus mas* L. and *Berberis vulgaris* fruit extracts at a concentration of 100 mg/mL, which were then mixed with a 50 mg/mL DPPH solution (0.004% methanolic). This mixture was kept in the dark at room temperature for 30 min [9]. The absorbance of the mixture was measured at 517 nm. Linearity ranges of DPPH were selected between 200 and 600 mmol/L using 5 calibration levels.

#### 2.4.2. Cuprac

In the CUPRAC method, copper (II)-neocuproin complex (Cu(II)-Nc) formed by 2.9-dimethyl-1, 10-phenanthroline (Neocuproin Nc) with Cu (II), copper antioxidant capacity is calculated by using the ability to reduce (I)-neocuproin (Cu(I)-Nc) chelate [10]. According to the method, 1 mL of each of 10 mM water-dissolved Cu (II), 7.5 mM neocuprine solution and 1 M pH 7.0 NH4Ac buffer was taken into the test tube and added to be the total volume, which was 4.1 mL from each of the samples prepared at different concentrations. The tubes were capped and left at room temperature for 1 h. Then, the absorbances of the mixtures against ethanol at 450 nm wavelength were read in Shimadzu UV-1800 UV-VIS Spectrophotometer. Each sample was run in 3 parallels and the results were given as the mean ± standard error of their absorbents. In addition, the A0.50 value, which is the concentration measured against A_0.50_ absorbance, is given as μg/mL [11]. Linearity ranges of CUPRAC were selected between 200 and 600 mmol/L using 5 calibration levels [7].

### 2.5. Cell line and samples preparation

In this study, breast cancer line MCF-7 cells were used to examine the proliferative and apoptotic effects of *C. mas* and *B. vulgaris* fruit extracts. MCF-7 cells stored at −196 °C in liquid nitrogen were rapidly thawed in a 37 °C water bath as soon as they were removed from the liquid nitrogen tank. Cells were counted with an automated cell counter (BioRad TC20) after passage and before seeding. Counting was performed by placing a counting slide on the device in accordance with the device manufacturer’s instructions. The dilution rate calculated by the instrument was used to achieve a concentration of 2.5 × 10^4^ cells/mL. Thus, 5 × 10^3^ cells were added to each well of the 96-well plate. After growing the cells in DMEM medium supplemented with 10% FBS and penicillin + streptomycin for 24 h, they were ready for the addition of the extracts.

### 2.6. Cytotoxicity measurement

On the breast cancer MCF-7 cell line, their cytotoxicity and IC_50_ values were determined by first studying them with 24-h and 48-h exposures over a wide range of dilution ratios. The extracts were added to the broth at 1:1, 1:2, 1:4, 1:8 and 1:16 dilution ratios in triplicate. After 24 h of incubation, the media were aspirated and removed from the cells that were ready. Extract and medium mixtures were prepared at the specified dilution ratios and the mixtures were added to the relevant wells. No extract was added to the cells in the control wells. Separate wells were prepared for 24-h and 48-h exposure. At the end of 24 and 48 h, XTT cytotoxicity test was performed for the relevant wells.

#### 2.6.1. XTT assay

After the cells were treated with the extracts, the reaction solution was obtained by mixing the XTT and the activation solution according to the kit manufacturer’s instructions. The XTT test was performed in 3 replicates for 1:1, 1:2 1:4, 1:8, and 1:16 dilutions and 2 incubation periods of 24 and 48 h. The mean value is calculated for three parallel samples corresponding to 1 extract. The obtained values are converted to cell viability relative to the control by rescaling the values. The noncytotoxic control is considered to have a relative viability of 100%. Values that result in relative cell viability values below 70% are deemed cytotoxic [12].

### 2.7. Cell death investigation by flow cytometry

Flow cytometric measurement was performed using Annexin-V/7-AAD commercial kit to investigate the role of *C. mas* and *B. vulgaris* fruits extracts in the breast cancer cell line. After XTT, dilution ratios were created according to the IC_50_ values, and the extracts were added after the MCF-7 cells multiplied by 70%–80% by inoculating into sterile 6-well plates. Cells were incubated for 24 and 48 h. At the end of the period, the Annexin-V/7-AAD kit procedure was performed. Apoptosis analysis was performed using the “Elabscience^®^ Annexin V-FITC Apoptosis Detection Kit”.

### 2.8. Statistical analysis

For cytotoxicity analysis on MCF-7 breast cancer, 24- and 48-h incubations were each performed in 3 replicates. The absorbance/fluorescence values of each replicate were evaluated by the microplate reader with the mean ± standard deviation table of mean cytotoxicity and viable cell % of all dilution ratios. Response curves for various dilution ratios of *C. mas* and *B. vulgaris* extracts were generated using two-way ANOVA analysis in Graphpad Prism 9.0.0 statistics software.

## 3. Results

The total phenolic substance content, total flavonoid substance amount and antioxidant capacity of the extracts and the extracted fruits were measured respectively by Folin Ciocalteu, spectrophotometric method and DPPH, CUPRAC extraction of *C. mas* and *B. vulgaris* fruits. The results are given in Table 1. While the total phenolic content of the *C. mas* fruit was 645.0 ± 11.8 mg GAE/100 g, the total phenolic content value of the extract was determined as 131.7 ± 6.0 mg GAE/L. While the total phenolic content of the *B. vulgaris* fruit was 966.3 ± 14.0 mg GAE/100 g, the total phenolic content value of the extract was determined to be 188.7 ± 5.0 mg GAE/L. While the total flavonoid content of the *C. mas* fruit was 11.2 ± 1.0 mg CE.100 g^−1^ dw, the total flavonoid content of the extract was 2.2 ± 0.1 mg CE.100 mL^−1^ dw. While the total flavonoid content of the *B. vulgaris* fruit was 721.0 ± 8.5 mg CE.100 mL^−1^ dw, the total flavonoid content of the extract was 141.3 ± 6.4 mg CE.100 mL^−1^ dw. While the DPPH radical scavenging power value of *C. mas* fruit was 1.2 ± 0.1 mmol TEAC/100 g, the extract value was determined to be 0.3 ± 0.0 mmol TEAC/mL. The DPPH radical scavenging power value of *B. vulgaris* fruit was 12.7 ± 0.1 mmol TEAC/100 g and the value of the extract was determined as 4.3 ± 0.3 mmol TEAC/mL. The Cu (II) ion reducing antioxidant capacity value of *C. mas* fruit was 2.2 ± 0.1 mmol/100g, whereas the value of the extract was 0.5 ± 0.0 mmol/mL. The value of the *B. vulgaris* Cu (II) ion reducing antioxidant capacity was 41.8 ± 1.6 mmol/100g, while the value of the extract was 7.8 ± 0.5 mmol/mL.

XTT test was applied to determine the effect of exposure of *C. mas* and *B. vulgaris* extracts to MCF-7 breast cancer cell line for 24 and 48 h on cancer cell viability. The cytotoxicity effect of both extracts was determined at 5 different dilutions as 1:1, 1:2 1:4, 1:8 and 1:16, and the % cell viability was calculated separately for each ratio. The cytotoxic effects of exposure with 24-h *C. mas* and *B. vulgaris* fruit extracts are given in Figure 1a. It is given as % viability due to increased concentration after 24 h in the MCF-7 cell line. It was determined that all dilution ratios of the studied *B. vulgaris* fruit extract had a significant cytotoxic effect against MCF-7 cancer cells. *C. mas* fruit extracts at 1:1, 1:2, 1:4 dilution ratios also showed significant cytotoxic effects against breast cancer cells. It has been determined that only at 1:8 and 1:16 extract dilution ratios does cell viability see a limited reduction (approximately 60%–70%) in healthy cells. The % vitality decreases as the concentration of and *C. mas* fruit extracts increases.

The cytotoxic effects of exposure to *C. mas* and *B. vulgaris* fruit extracts for 48 h are given in Figure 1b. It is given as % viability due to increased concentration in the MCF-7 cell line after 48 h. It was determined that all dilution ratios of the studied *B. vulgaris* fruit extract had a significant cytotoxic effect against MCF-7 cancer cells. *C. mas* fruit extracts at 1:1, 1:2, 1:4 dilution ratios also showed significant cytotoxic effects against breast cancer cells. However, it was determined that *C. mas* fruit extracts at 1:8, 1:16 dilution ratios did not show cytotoxic activity against breast cancer cells. The percentage of vitality decreases as the concentration of *Berberis vulgaris* and *Cornus mas* L. fruit extracts increases.

The IC_50_ values of *C. mas* and *B. vulgaris* fruit extracts in MCF-7 cells according to the XTT results after 24 and 48 h of exposure are shown in Table 2. The 24 h IC_50_ value of *C. mas* and *B. vulgaris* fruit extracts in MCF-7 cells was calculated as 1:43.47 and 1:22 dilution ratios, respectively. The 48 h IC_50_ value of the extracts in MCF-7 cells was calculated as 1:21.24 and 1:10 dilution ratio, respectively. In the MCF-7 cell line, 48 h of incubation was more effective than 24 h.

The apoptotic activities of the cells were analyzed by making dilution ratios according to the specified IC_50_ values. Consequently, *C. mas* extract was diluted 1:10, 1:20, 1:40, 1:80 and *B. vulgaris* extract was diluted 1:5, 1:10, 1:20, 1:40 after being applied to cells, then incubated for 24 and 48 h. The results were evaluated by comparing with the control groups to which the extracts were not added.

Data on the mean and standard deviations of viable cell count, early apoptotic cell count, late apoptotic cell count, and necrotic cell count at all dilution ratios of MCF-7 cells exposed to *C. mas* extract and *B. vulgaris* extract for 24 h are shown in Table 3. According to the results obtained, cell viability of MCF-7 cells exposed to *C. mas* extract for 24 h at all dilution ratios was found to be significantly lower when compared to the control group (p < 0.0001). While the percentage of viable cells in the control group was 94.48 ± 3.11, the percentage of viable cells at 1:10 and 1:20 dilution ratios were 37.83 ± 2.5 and 45.27 ± 3.52, respectively. Likewise, the number of early apoptotic cells was found to be significantly higher in all dilution ratios compared to the control group (p < 0.0001). While the percentage of early apoptotic cells in the control group was 2.13 ± 1.17, the percentage of early apoptotic cells at 1:40 and 1:80 dilution ratios were found to be 42.28 ± 13.5 and 32.92 ± 9.46, respectively. The number of late apoptotic cells at 1:10 dilution ratio of MCF-7 cells exposed to *C. mas* extract for 24 h was significantly higher (p = 0.0021). While the percentage of late apoptotic cells in the control group was 3.0 ± 3.93, the percentage of late apoptotic cells in the 1:10 dilution ratio was found to be 25.01 ± 15.1. However, no significant difference was found in terms of the number of necrotic cells (p > 0.05). According to the results, cell viability of MCF-7 cells exposed to *B. vulgaris* extract for 24 h at all dilution ratios was found to be significantly lower when compared to the control group (p < 0.0001). At the same time, the number of early apoptotic cells at 1:5 dilution ratio was found to be significantly higher when compared to the control group (p = 0.01). The number of early apoptotic cells at the 1:10 dilution ratio was also significantly higher when compared to the control group (p = 0.0004), as was the number of early apoptotic cells at 1:20 dilution rate (p = 0.0094). While the percentage of early apoptotic cells in the control group was 2.13 ± 1.17, the percentage of early apoptotic cells at 1:5 and 1:10 dilution ratios were found to be 30.1 ± 25.7 and 40.44 ± 3.44, respectively. The percentage of late apoptotic cells in all dilution ratios of MCF-7 cells exposed to *B. vulgaris* extract for 24 h was found to be significantly higher when compared to the control group. MCF-7 cells exposed to *C. mas* extract for 24 h showed significantly higher number of late apoptotic cells at 1:5 and 1:20 dilution ratios (p < 0.0001). The number of late apoptotic cells at 1:40 dilution ratio of MCF-7 cells exposed to *C. mas* extract for 24 h was significantly higher (p = 0.0118). While the percentage of late apoptotic cells in the control group was 3.0 ± 3.93, the percentage of late apoptotic cells at 1:5 dilution ratio was found to be 58.93 ± 29.74. However, no significant difference was found in terms of the number of necrotic cells.

Data on the mean and standard deviations of viable cell count, early apoptotic cell count, late apoptotic cell count, and necrotic cell count at all dilution ratios of MCF-7 cells exposed to *C. mas* extract and *B. vulgaris* extract for 48 h are shown in Table 4. The results indicate that cell viability of MCF-7 cells exposed to *C. mas* extract for 48 h at all dilution ratios was significantly lower when compared to the control group (p < 0.0001). The percentage of late apoptotic cells in all dilution ratios of MCF-7 cells exposed to *C. mas* extract for 48 h was found to be significantly higher when compared to the control group. The number of late apoptotic cells at 1:10 dilution ratio was found to be significantly higher when compared to the control group (p = 0.0018). The number of late apoptotic cells at 1:20 dilution ratio was found to be significantly higher when compared to the control group (p = 0.0014). The number of late apoptotic cells at 1:40 dilution ratio was significantly higher when compared to the control group (p = 0.0003). The number of late apoptotic cells at 1:80 dilution ratio was significantly higher when compared to the control group (p < 0.0001). However, no significant difference was found in terms of the number of early apoptotic cells and the number of necrotic cells (p > 0.05). Cell viability of MCF-7 cells exposed to *B. vulgaris* extract for 48 h at all dilution ratios was found to be significantly lower when compared to the control group. Cell viability at 1:5 and 1:10 dilution ratios was significantly lower when compared to the control group (p < 0.0001). Cell viability at 1:20 dilution ratio was found to be significantly lower when compared to the control group (p = 0.0034). Cell viability at 1:40 dilution ratio was found to be significantly lower when compared to the control group (p = 0.0262). The number of early apoptotic cells at 1:5 dilution ratio was found to be significantly higher when compared to the control group (p = 0.0024). The number of early apoptotic cells at the 1:10 dilution ratio was significantly higher when compared to the control group (p < 0.0001). While the percentage of early apoptotic cell number in the control group was 2.13 ± 1.17, the percentage of early apoptotic cell number at 1:10 and 1:20 dilution ratios was found to be 24.56 ± 11.63 and 8.39 ± 0.81, respectively. The percentage of late apoptotic cells at 1:5 and 1:10 dilution ratios of MCF-7 cells exposed to *B. vulgaris* extract for 48 hours was found to be significantly higher compared to the control group (p < 0.0001, p = 0.0002). While the percentage of late apoptotic cell count in the control group was 3.0 ± 3.93, the percentage of early apoptotic cell count at 1:5 and 1:10 dilution ratios was 79.35 ± 5.64 and 20.05 ± 10.46, respectively. There was no significant difference in the number of necrotic cells in MCF-7 cells exposed to *B. vulgaris* extract for 48 h (p > 0.05).

## 4. Discussion

This study was carried out to examine the effects of extracts from *Cornus mas* L. and *Berberis vulgaris* fruits on the proliferation and apoptosis of MCF-7. In recent years, many studies have provided important information regarding the anticancer properties of fruits. Some fruits, particularly those that contain flavonoids and other bioactive compounds, have been shown to have anticancer effects, such as inhibiting the growth of cancer cells and triggering the pathway of apoptosis. Therefore, the fact that fruits are rich in antioxidant and anticancer compounds plays an important role in the prevention and treatment of cancer, making them a valuable component of a healthy diet. To deduce the anticancer properties of the fruits used in this study, they were examined for their flavonoid content, antioxidant capacity, and phenolic compound content. Today, attention has been drawn to the anticancer activity of flavonoids. It seems that the anticancer effect of flavonoids is mainly due to their antioxidant and antiinflammatory activities, and their ability to modulate molecular signaling pathways involved in cell survival, proliferation, and mutation [13]. Flavonoids, a large group of phenolic compounds with beneficial activities for human health, are among the few important components of edible wild fruits. In this study, the total amount of flavonoid substances ranged from 2.2 ± 0.1 mg/mL to 721.0 ± 8.5 mg/100g, showing significant differences between species. When the species we studied in this study were compared, it was found that the total flavonoid content of the raw *B. vulgaris* fruit was 65.5 times higher than the raw version of the *C. mas* fruit. In the extracted form of the studied species, the total amount of flavonoid substances of *B. vulgaris* extract was found to be 64.2 times higher than that of *C. mas* extract. In a study conducted to determine the chemical and phytochemical contents of *B. vulgaris* genotypes collected from the Sivaslı district of Uşak, it was stated that the total flavonoid substance amounts of 16 different genotypes of *B. vulgaris* fruit ranged from 261.66 to 965.97 mg/mL [14]. In the study conducted by Awan et al. in terms of nutritional and medicinal contents of *B. vulgaris* fruits in Pakistan, they reported that the total amount of flavonoids in *B. vulgaris* fruits ranged from 376.93 to 395.09 mg/mL [15]. In this study, the total flavonoid content of *B. vulgaris* fruits was found to be 721.0 ± 8.5, while the total flavonoid content of the extract was 141.3 ± 6.4 mg CE.100 g^−1^ dw. The results we achieved in this study contain data that is similar data to that of the literature. In a study examining the antioxidant properties of different types of *C. mas* extracts collected from Malatya, the total flavonoid content of acetone extract was found to be 255.75 ± 14.92 mg CE.100 mL^−1^dw, and the total amount of flavonoids of methanol extract was 80.54 ± 4.71 mg CE.100 mL^−1^ dw [16]. In a study examining the phenolic acid, flavonoid profiles, and antioxidant properties of extracts from edible wild fruits, the total flavonoid content of *C. mas* methanol extracts was discovered to be 17.27 ± 0.8 mg CE.100 mL^−1^ dw [17]. The total flavonoid content can be influenced by factors such as genetic factors and environmental conditions as well as the degree of ripeness of the fruit, the harvesting method, processing methods, and storage conditions. Such as, immature fruits have less flavonoids, and the time of harvesting and storage conditions can also affect the flavonoid content. Additionally, the climate and soil characteristics of the region where the fruits are grown are also factors that can affect the flavonoid content. In this study, the total flavonoid content of *C. mas* extract was 2.2 ± 0.1 mg CE.100 mL^−1^dw. Phenolic compounds exhibit antioxidant activity, antiinflammatory effects, and antiproliferative effects [18]. In this study, significant differences were found between *B. vulgaris*, *C. mas* fruits and fruit extracts analyzed by the Folin-Ciocalteu method in terms of total phenolic content. According to our results, the quantity of phenolic substances in *B. vulgaris* fruit is 1.5 times higher than in *C. mas* fruit. In the process of converting these fruits into extracts, the amount of phenolic substances decreased at a similar rate, approximately 5 times. In a study examining the antioxidant activities and phenolic compounds in *C. mas* fruit varieties collected from Ukraine, the results of the total phenolic content of the fruits determined by the Folin-Ciocalteu method ranged from 91.34 to 289.79 mg/100 g [19]. In another study conducted with *C. mas* collected from Romania, the total phenolic content of the fruits was reported to be between 163.69 ± 0.04 and 359.28 ± 9.57 mg/100 g [20]. Research aiming to protect the sensory and nutritional quality of *C. mas* fruits during post-harvest cold storage by delaying softening and increasing phenol accumulation with γ-aminobutyric acid and nitric oxide applications noted that the total phenolic substance content was 611.6 ± 17.79 to 988.3 ± 36.1 mg. /100g [21]. Another study that was aiming to determine the content of phenolics in the fruits of *C. mas* species by spectrophotometric methods found the total phenolic content of the water extract to be 12.77 ± 0.81 mg/g [22]. In this study, the total phenolic content of *C. mas*, a member of the Cornus species, was found to be 645.0 ± 11.8 mg/100g. Compared to the values reported in the literature, the total phenolic content of the *C. mas* fruit utilized in this study appears to be relatively high. The quantity of phenolic substances can be affected by factors such as the climate, soil, stress conditions, storage conditions, and lifespan of the fruit [23]. The variation in the number of phenolic substances in *C. mas* fruit can be attributed to this reason. In a study conducted to determine the highest total amount of phenolic compounds in different extraction methods, *C. mas* extract phenolic content value in the ultrasonic extraction method was found to be 131.31 mg/mL [24]. In this study, the total phenolic content of *C. mas* extract was found to be 131.7 ± 6.0 mg/mL, a result similar to what can be found in the literature. The total phenolic content of *B. vulgaris*, another fruit used in this study, which is a member of the Berberis species, was found to be 966.3 ± 14.0 mg/100g. In a study on the quality of *B. vulgaris* juice obtained by direct blender of *B. vulgaris* fruit, the total phenolic content of the fruit juice was 992.18 ± 8.95 mg/100g [25]. The total phenolic content value of the extract of *B. vulgaris* fruit was determined to be 188.7 ± 5.0 mg/mL. In a study conducted to examine the effect of *B. vulgaris* fruit in patients with primary biliary cholangitis who did not respond to treatment, the total phenolic content of *B. vulgaris* extract was calculated to be 105 ± 12 mg/mL [26]. In another study, *B. vulgaris* extract was evaluated in terms of total phenolic content and the obtained value was 100.862 ± 1.967 mg/mL [27]. Research conducted in the Sivas province of Turkey noted the total phenolic component content of fresh fruits that were collected and immediately processed ranged from 256.5 to 362.9 mg/mL [28]. In 2009, the amount of phenolic compounds for *B. vulgaris* fruit extracts collected from Malaysia was 100 mg/mL for water extract and 280 mg/mL for methanolic extract, respectively [29]. The results we reached in this study contain data comparable to that in the literature. The results of total phenolic content show that *B. vulgaris* may be one of the richest sources of phenolic compounds. This study investigated the total phenolic content of fruits and extracts of *Cornus mas* L. and *Berberis vulgaris* collected from the same region. The results showed a significant difference in total phenolic content between the two species, despite having grown within the same ecological conditions. One possible explanation for this difference could be attributed to genetic factors. However, it is also possible that the difference in total phenolic content could be due to the different abilities of the two species to adapt to changing environmental conditions. Further research is needed to understand the underlying mechanisms of this difference and how it affects the health benefits of these fruits [30].

A previous study conducted on *Cornus mas* L. fruits collected from Malatya analyzed the antioxidant capacities of acetone extract using the DPPH method. The results of the study reported the antioxidant properties of the fruits as 1.053 ± 38.1 mg/100 g [16]. Additionally, another study reported the antioxidant activities of 6 different *Cornus mas* L. cultivars using the DPPH method, and the results ranged from 1.24 to 2.71 mmol/100 g [17]. Our study also utilized the DPPH method to analyze the total antioxidant capacity of *Cornus mas* L. fruit and its extract, yielding results of 1.2 ± 0.1 mmol/100g and 0.3 ± 0.0 mmol/L, respectively. These findings align with data reported in the literature. In a comparative study on the antioxidant potentials of *C. mas*, fruits were collected from nature in Susurluk district of Balıkesir and the antioxidant capacity was determined to be 20.9 ± 0.1 mgTE/g using the CUPRAC method [31]. In a study comparing the phytochemical compositions and antioxidant capacities of 3 different cherry varieties, the antioxidant capacity of *C. mas* fruit was analyzed by the CUPRAC method, and the result was reported to be 76.3 ± 3.6 mgTE/g [32]. In this study, the results of the analysis of the total antioxidant capacity of the *C. mas* fruit and its extract, determined by the CUPRAC method, were found to be 2.2 ± 0.1 mmol/100g and 0.5 ± 0.0 mmol/L, respectively. The results of our study on the antioxidant properties of *Cornus mas* L. and *Berberis vulgaris* fruits and extracts are consistent with previous literature. In a study conducted in Bayburt, Türkiye to analyze the physicochemical properties, antioxidant capacities, phenolic compound profiles, and antimicrobial activities of naturally grown *Berberis vulgaris* fruit, the total antioxidant capacity of the extracts was measured by the DPPH method. The results ranged from 11.92 ± 0.22 to 40.44 ± 1.99 mmol/L [33]. Similarly, our study found that the total antioxidant capacity of the *Berberis vulgaris* extract was 16.2 ± 1.1 mmol/L. In a review article on the polyphenol content and antioxidant capacity of grape species, it was stated that the total antioxidant capacity of *B. vulgaris* fruit ranged from 8.731 to 90.63 mmol/100 g [34]. The analysis results of the total antioxidant capacity of the *B. vulgaris* fruit and the extract of the fruit used in this study, determined by the DPPH method, were found to be 12.7 ± 0.1 mmol/100g and 4.3 ± 0.3 mmol/L, respectively. In general, the number of phenolic components, flavonoids, and antioxidant capacity measured in *Berberis vulgaris* are higher than those of *Cornus mas* L. Additionally, the antioxidant activity of *Berberis vulgaris* fruit is higher than that of *Cornus mas* L. fruit, as determined by both the DPPH and CUPRAC methods. Particularly, the DPPH method clearly shows a difference in antioxidant activity even between varieties of the same fruit [35]. Moreover, it is crucial for the antioxidant activity method to reflect the physiological conditions. In this study, the total amount of phenol obtained was higher than the findings of the aforementioned studies. This may be due to variations in the ripening stages of the fruits or other environmental factors [36]. Nevertheless, the results of this study are consistent with the existing literature.

In this study, *B. vulgaris* fruit extracts reduced the viability of MCF-7 cells after 24 and 48 h of exposure in a dilution-dependent manner. Inhibition of cell viability by *B. vulgaris* extracts is stronger than the effect of *C. mas* extract at 24 h and 48 h. This significant difference can be explained by the increased phenolic compound and antioxidant capacity, as well as the higher sensitivity of the breast cancer cell line to *B. vulgaris* extract compared to *C. mas* extract [37]. The stimulant effect of *B. vulgaris* extract on cell viability is unusual and should be questioned. Although similar results are found in the literature, they can be rejected for a few reasons. We observed that the cell density was evenly distributed among the samples prior to the addition of the extracts. Therefore, the measured viability values cannot be related to the different initial cell densities between samples [38]. Therefore, *B. vulgaris* extract may have produced a real stimulatory effect in the MCF-7 cell line. The reason for the higher percentages of viability at 48 h of exposure to *B. vulgaris* extract at the same dilution ratios for 24 h and 48 h may be attributed to higher energy-consuming activities prior to apoptosis. A previous study suggested that cells try to compensate for the increased apoptotic rate by amplifying cell proliferation [39]. In a study showing anticancer activity of *B. vulgaris* fruit extract in the MCF-7 cell line, the extract reduced cell proliferation in a time- and dose-dependent manner. Different extraction processes were applied, and ethanol extract was found to be more active than water extract, probably due to its capacity to remove more compounds responsible for anticancer activity, such as alkaloids [40]. The main biological feature of cancer cells is uncontrolled and rapid proliferation, so tumor growth arrest is considered a viable treatment option for cancer. The anti-proliferative effect of *B. vulgaris* has been recognized in various cancers, including breast, liver, and colon cancers, but its molecular mechanisms of action have not yet been established [41–42]. In this study, it was observed that *C. mas* fruit extracts decreased the viability of MCF-7 cells after 24 and 48 h of exposure, depending on the dilution ratio. In breast cancer cells, no cytotoxic mechanism of action was found at 1:8 and 1:16 dilution ratios (at lower concentrations) of *C. mas* extract in 48 h. Depending on the dilution rate, inhibition became apparent only at higher concentrations. In the literature, cytotoxic studies with *C. mas* are limited to tumor cells only, the effects of extracts on the viability of noncancerous cells have not been evaluated yet [43]. In one study, the aqueous extract of *C. mas* exhibited cytotoxicity against MCF-7 cells at a high dose of 750 μg/mL [44]. In a study examining the antitumor effect of *C. mas* fruit in Balb/C mice, *C. mas* extract was found to strongly inhibit cancer proliferation in vitro [45]. Consistent with the literature, the data of this study showed that *B. vulgaris* and *C. mas* extract significantly reduced breast cancer cell proliferation in a time- and dose-dependent manner. The IC_50_ value represents inhibitory concentrations and is used at the beginning of the study to evaluate the suitability/performance of drugs [46]. The IC_50_ value can be defined as the minimum extract concentration value to reduce cell viability to 50%. The fruit extracts in this study reduced the viability of MCF-7 cells in a dilution-dependent manner after 24 and 48 h of exposure. The IC_50_ values of *B. vulgaris* and *C. mas* fruit extract at 24 h were 4.5% and 2.3%, respectively. The IC_50_ values of *B. vulgaris* and *C. mas* fruit extract at 48 h were 4.7% and 1%, respectively. The IC_50_ values strongly indicated that effective doses of *B. vulgaris* fruit extracts were higher after different incubation times (24–48 h) compared to *C. mas* fruit extracts. Therefore, *B. vulgaris* fruit extract inhibited the growth of MCF-7 cells more effectively than *C. mas* fruit extract. For over 3 decades, the mainstay and goal of clinical oncology has been the development of therapies that promote the effective elimination of cancer cells through apoptosis. This process of programmed cell death is mediated by several signaling pathways (called intrinsic and extrinsic) triggered by many factors, including cellular stress, DNA damage, and immune surveillance. Interaction of apoptosis pathways with other signaling mechanisms may also affect cell death. Tumor cell death may result in response to therapy, while selection, growth, and spread of resistant cells may ultimately be fatal [47].

Inducing cell apoptosis is an ideal way to kill cancer cells. In this study, breast cancer cells underwent apoptosis when treated with *B. vulgaris* and *C. mas* extracts for 24 and 48 h. Breast cancer cells were exposed to higher, closer, and lower dilution rates than IC_50_ values. Annexin V-FITC/ 7AAD dual staining on breast cancer cells treated with *B. vulgaris* extract for 24 and 48 h were analyzed by flow cytometry to examine the inhibition mechanism of measles extract on the proliferation of cells. The results showed that *B. vulgaris* fruit extract could significantly promote MCF-7 cell apoptosis compared to the control group at all dilution rates at 24 and 48 h (p < 0.0001). According to the findings of this study, the number of viable cells decreased as the concentration of *B. vulgaris* fruit extract increased. It increased early and late apoptosis in MCF-7 cells depending on the increasing concentration of *B. vulgaris* extract (p < 0.05). In a study examining the antioxidant properties of *B. vulgaris* and its cytotoxic effect on human breast cancer cells, it was shown that *B. vulgaris* has strong antioxidant properties and cytotoxic effects that can induce apoptosis [40]. In another study examining the apoptotic pathways of *B. vulgaris* extract in hepatic cells, antiproliferative and proapoptotic effects were evident upon suppressing caspase-3 activation and lipid accumulation [48]. In another study, *B. vulgaris* exhibited potent anticancer activities with the induction of apoptosis in esophageal carcinoma cells [49]. In a study evaluating the anticancer properties of *B. vulgaris* on MCF-7 and T47D breast cancer cell lines, *B. vulgaris* alone inhibited cell proliferation and induced apoptosis [50]. The results of this study are compatible with the literature.

Annexin-V/7-AAD dual staining on breast cancer cells treated with *C. mas* fruit extract for 24 and 48 hours were analyzed by flow cytometry to examine the inhibition mechanism of *C. mas* extract on the proliferation of cells. The results showed that *C. mas* extract could significantly promote MCF-7 cell apoptosis compared to the control group at all dilution rates at 24 and 48 h (p < 0.0001). According to the findings of this study, the number of viable cells decreased as the concentration of *C. mas* fruit extract increased. At 24 h, *C. mas* extract significantly increased early apoptosis in MCF-7 cells due to increasing concentration (p < 0.0001). At 48 h, *C. mas* extract significantly increased late apoptosis in MCF-7 cells due to increasing concentration (p < 0.05). A study examining the induction of apoptosis of *C. mas* extract in a gastric cancer cell line concluded that *C. mas* acts as a new, potent inhibitor of cancer proliferation [51]. Research evaluating antioxidant and antiproliferative properties of *C. mas* juice found that *C. mas* juice showed an antiproliferative effect against CT26 colon cancer cells and HepG2 liver cancer cells [52]. Although there is data on the cytotoxic activity of *C. mas* extracts against cancer cells in the literature, there are not many studies on their in vitro antiproliferative properties [53].

This study demonstrated that acetone extracts of *Berberis vulgaris* and *Cornus mas* L. fruits induce apoptosis in MCF-7 breast cancer cells. The cell death-inducing effects of the acetone extracts were determined using fluorescent microscopy and flow cytometry methods. The results show that the acetone extracts of *Berberis vulgaris* and *Cornus mas* L. fruits have potential therapeutic effects in inducing apoptosis in MCF-7 cells. Extracts obtained from both *Cornus mas* L. and *Berberis vulgaris* fruits had similar potential for inducing cell death. According to IC_50_ values, extracts obtained from *Berberis vulgaris* fruits showed stronger apoptotic effects in MCF-7 cells. Extracts obtained from *Cornus mas* L. fruits can also induce cell death at high concentrations. These results demonstrate that acetone extracts of *Berberis vulgaris* and *Cornus mas* L. fruits have potential therapeutic effects not only in MCF-7 breast cancer cells but also in normal cells. The results of this study indicate that further investigation of acetone extracts obtained from *Berberis vulgaris* and *Cornus mas* L. fruits is warranted. In conclusion, acetone extracts obtained from *Berberis vulgaris* and *Cornus mas* L. fruits can be evaluated as potential anticancer agents.

This study demonstrates the potential of acetone extracts obtained from *Berberis vulgaris* and *Cornus mas* L. fruits to induce apoptosis in MCF-7 breast cancer cells. However, the study has certain limitations. These limitations include the fact that it is an in vitro study conducted under laboratory conditions, only one cell line was used, evaluation of the in vivo toxicity of acetone extracts did not occur, and the characterization of the chemical components of the extracts was incomplete. Among the strongest aspects of this study is the significant potential of acetone extracts obtained from *Berberis vulgaris* and *Cornus mas* L. fruits to trigger apoptosis in MCF-7 breast cancer cells. Additionally, the study has ensured the reliability and comparability of results by employing standardized experimental protocols. The findings suggest that these fruit extracts hold promise for potential use in cancer treatment, laying the foundation for further drug development efforts. Finally, the study underscores the importance of exploring the potential positive effects of natural sources in cancer treatment.

## Figures and Tables

**Figure 1a:**
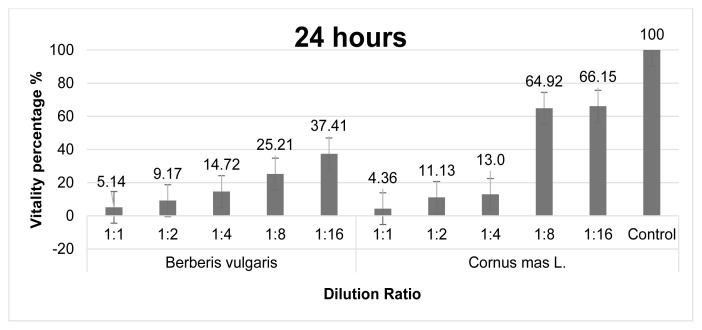
Graph of % viability of *B. vulgaris* and *C. mas* extracts after 24 h in the MCF-7 cell line.

**Figure 1b:**
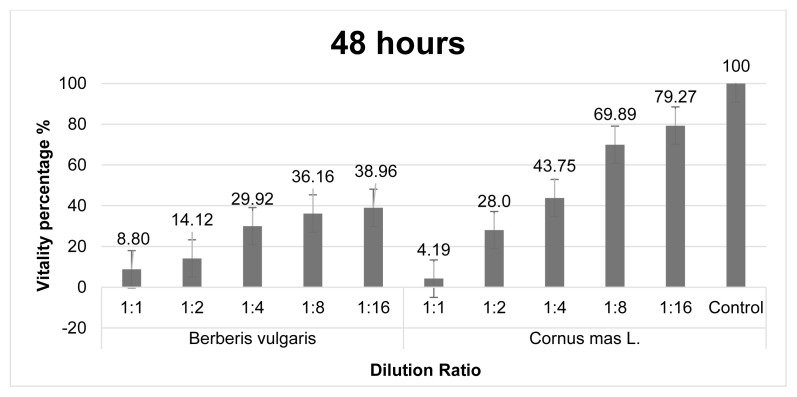
Graph of % viability of *B. vulgaris* and *C. mas* extracts after 48 h in MCF-7 cell line.

**Table 1:** Total phenolic content, total flavonoid content, antioxidant capacity CUPRAC and DPPH results of *C. mas* and *B. vulgaris* berries and their extracts.

**Total amount of phenolic substanc**e		
**Fruit samples**	Fruit form GAE/100 g	Extract form mg/L GAE
***C. mas*** ** (** ***Cornus mas*** ** L.)**	645.0 ± 11.8	131.7 ± 6.0
***B. vulgaris***	966.3 ± 14.0	188.7 ± 5.0
**Total flavonoid content**		
**Fruit samples**	Fruit form mg/CE.100 g^−1^	Extract form mg/CE.100 mL^−1^
***C. mas*** ** (** ***Cornus mas*** ** L.)**	11.2 ± 1.0	2.2 ± 0.1
***B. vulgaris***	721.0 ± 8.5	141.3 ± 6.4
**Antioxidant capacity DPPH radical scavenging power**		
**Fruit samples**	Fruit form mmol/100 g	Extract form mmol/L
***C. mas*** ** (** ***Cornus mas*** ** L.)**	1.2 ± 0.1	0.3 ± 0.0
***B. vulgaris***	12.7 ± 0.1	4.3 ± 0.3
**Cu(II) ion reducing antioxidant capacity**		
**Fruit samples**	Fruit form mmol/100 g	Extract form mmol/L
***C. mas*** ** (** ***Cornus mas*** ** L.)**	2.2 ± 0.1	0.5 ± 0.0
***B. vulgaris***	41.8 ± 1.6	7.8 ± 0.5

**Table 2:** IC_50_ values of *C. mas* and *B. vulgaris* extracts.

IC_50_ values		
Fruit samples	24 h	48 h
	Dilution rate	Dilution rate
***C. ma*** **s**	1:43.47	1:10
***B. vulgaris***	1:22	1:21.24

**Table 3:** 24-h Annexin-V/7-AAD analysis of *C. mas* and *B. vulgaris* extract in breast cancer cell line % cell ratios (n = 3).

	Number of living cells (%)	Number of early apoptotic cells (%)	Number of late apoptotic cells (%)	Number of necrotic cells (%)
***C. mas*** ** extract Dilution rate**				
**1:10**	37.83 ± 2.5*	36.47 ± 13.82*	25.01 ± 15.1*	0.69 ± 0.4
**1:20**	39.55 ± 10.1*	42.01 ± 10.45*	14.5 ± 0.55	0.6 ± 0.9
**1:40**	45.27 ± 3.52*	42.28 ± 13.5*	12.04 ± 6.16	0.03 ± 0.04
**1:80**	55.32 ± 4.45*	32.92 ± 9.46*	11.73 ± 5.02	0.02 ± 0.01
**Control**	94.48 ± 3.11	2.13 ± 1.17*	3.0 ± 3.93	0.4 ± 0.16
	**p < 0.0001** *	**p < 0.0001** *	**p = 0.0021** *	
***B. vulgaris*** ** extract dilution rate**				
**1:5**	10.7 ± 4.81*	30.1 ± 25.7*	58.93 ± 29.74*	0.3 ± 0.23
**1:10**	27.5 ± 8.16*	40.44 ± 3.44*	32.04 ± 10.87*	0.53 ± 0.05
**1:20**	23.23 ± 13.4*	30.45 ± 4.21*	46.10 ± 17.32*	0.23 ± 0.15
**1:40**	50.12 ± 3.33*	18.09 ± 4.37	30.58 ± 4.5*	0.86 ± 0.68
**Control**	94.48 ± 3.11	2.13 ± 1.17	3.0 ± 3.93	0.4 ± 0.16
	**p < 0.0001** *	**p < 0.05** *	**p < 0.05** *	

* Significantly changed compared to the positive control.

**Table 4:** 48-h Annexin-V/7-AAD analysis to *C. mas* extract and *B. vulgaris* extract in breast cancer cell line % cell ratios (n = 3).

	Number of living cells (%)	Number of early apoptotic cells (%)	Number of late apoptotic cells (%)	Number of necrotic cells (%)
***C. mas*** ** extract dilution rate**				
**1:10**	80.0 ± 3.85*	5.14 ± 1.6	9.44 ± 2.13*	2.63 ± 1.06
**1:20**	83.46 ± 1.76*	4.7 ± 1.31	9.62 ± 1.01*	2.28 ± 0.7
**1:40**	83.64 ± 1.21*	4.56 ± 0.52	10.6 ± 0.83*	1.26 ± 0.14
**1:80**	82.8 ± 3.53*	5.83 ± 0.8	12.33 ± 3.9*	1.87 ± 0.56
**Control**	94.48 ± 3.11	2.13 ± 1.17	3.0 ± 3.93	0.4 ± 0.16
	**p < 0.0001** *		**p < 0.05** *	
	**Number of living cells (%)**	**Number of early apoptotic cells (%)**	**Number of late apoptotic cells (%)**	**Number of necrotic cells (%)**
***B. vulgaris*** ** extract dilution rate**				
**1:5**	3.00 ± 1.26*	15.77 ± 4.3*	79.35 ± 5.64*	1.87 ± 0.11
**1:10**	54.22 ± 4.82*	24.56 ± 11.63*	20.05 ± 10.46*	1.17 ± 0.8
**1:20**	81.28 ± 1.35*	8.39 ± 0.81	9.47 ± 1.07	0.85 ± 0.29
**1:40**	84.13 ± 5.1*	4.8 ± 0.4	10.55 ± 5.5	0.56 ± 0.07
**Control**	94.48 ± 3.11	2.13 ± 1.17	3.0 ± 3.93	0.4 ± 0.16
	**p < 0.05** *		**p < 0.05** *	

* Significantly changed compared to the positive control.
